# Dentin Biomodification with Flavonoids and Calcium Phosphate Ion Clusters to Improve Dentin Bonding Stability

**DOI:** 10.3390/ma15041494

**Published:** 2022-02-17

**Authors:** Youna Paik, Jae-Hoon Kim, Kyung-Hyeon Yoo, Seog-Young Yoon, Yong-Il Kim

**Affiliations:** 1 Department of Orthodontics, Dental Research Institute, Pusan National University, Yangsan 50612, Korea; ynplove@naver.com; 2 Department of Dental Education, Dental Research Institute, Pusan National University, Yangsan 50612, Korea; jhkimdent@pusan.ac.kr; 3 Dental and Life Science Institute, Pusan National University, Yangsan 50612, Korea; 4 School of Materials Science and Engineering, Pusan National University, Busan 46241, Korea; seweet07@pusan.ac.kr (K.-H.Y.); syy3@pusan.ac.kr (S.-Y.Y.)

**Keywords:** cross-linking agents, dentin bond strength, flavonoids, remineralization

## Abstract

The purpose of this study was to evaluate the effects of flavonoids and calcium phosphate ion clusters (CPIC) on dentin bonding stability. Seven experimental solutions were synthesized using icaritin (ICT), fisetin (FIS), silibinin (SIB), CPIC, and combinations of one of three flavonoids and CPIC (ICT + C, FIS + C, SIB + C). The experimental solutions were applied to demineralized dentin prior to the application of a universal adhesive. A group without any experimental solution served as a control. Dentin specimens pretreated with the experimental solutions were assayed using Fourier transform infrared (FTIR) spectroscopy. The microtensile bond strength (µTBS) and nanoleakage were evaluated at 24 h and after 10,000 thermocycles. FIS and ICT + C showed significantly higher µTBS than the control group at 24 h. CPIC, ICT + C, FIS + C, and SIB + C showed significantly higher µTBS than the control group after thermocycling. After thermocycling, silver infiltration into the hybrid layer and interfacial gaps was more noticeable in the control group than in the other groups. The FTIR spectra revealed the formation of apatitic minerals in the demineralized dentin in the flavonoid and CPIC combination groups. The pretreatment of demineralized dentin with flavonoids and CPIC improved dentin bonding stability. The flavonoid and CPIC combinations preserved dentin bond strength.

## 1. Introduction

Despite continuous advancements in dentin bonding systems, resin-dentin interfaces are still the most vulnerable part of adhesive restorations [1,2]. The longevity of adhesive restorations depends on the stability of the so-called hybrid layer, which consists of resin penetrating into the collagenous network of the dentin [3]. The bond strength of adhesion to dentin also relies mostly on the hybrid layer. However, collagen fibrils that are exposed during the bonding procedure are not completely infiltrated by resin monomers [4,5]. An incomplete infiltration leaves unprotected collagen and porosities in the hybrid layer. This phenomenon was termed “nanoleakage”, which can work as the pathway for the degradation of resin-dentin bonds over time. Unprotected collagen is prone to degradation caused by acidic products of oral bacteria such as Streptococcus mutans and hydrolysis, associated with the collagenolytic enzymes, such as matrix metalloproteinases (MMPs) [2,6,7,8]. The degradation of the collagenous matrix at resin-dentin interfaces impairs dentin bonding stability, which results in a decrease in bond strength and increase in nanoleakage.

Many attempts have been made to improve the stability of resin-dentin interfaces by inhibiting biodegradation and improving the mechanical properties of the hybrid layer [9,10,11]. A new strategy to improve the stability and longevity of dentin bonding is to inhibit MMPs released by dentinoblasts within the collagen fiber network of dentin [7]. This enzymatic inactivation method uses an MMP inhibitor before adhesive application to demineralized dentin. Flavonoids, which are natural cross-linking agents, are also MMP inhibitors [12]. They are a large family of over 5000 hydroxylated polyphenolic compounds that perform important functions in plants, including combating environmental stresses, such as microbial infection, and regulating cell growth [13]. Several flavonoids were used to improve dentin bonding stability [12,14,15,16]. Hesperidin, a flavonoid extracted from citrus fruits, improves the mechanical properties of resin-dentin interfaces, and increases the immediate bonding strength of a self-etching adhesive [12]. Quercetin, rutin, and naringin were proven to be effective for improving the long-term stability of dentin bonding [16]. However, there is a lack of studies on the effects of various types of flavonoids in dentin bonding.

Another strategy to improve dentin bonding stability is to replace the residual water in non-resin-encapsulated demineralized collagen fibers with new apatite minerals [11]. The infiltration of resin monomers into the demineralized dentin matrix to form a sound hybrid layer is the key to achieving durable resin-dentin bonds [2,10]. However, a discrepancy between the depth of demineralization and resin infiltration is unavoidable. Unprotected dentin collagen fibers are vulnerable to hydrolysis and enzymatic degradation [10]. The formation of apatitic minerals in the demineralized dentin matrix can prevent collagen fibers from being degraded by collagenolytic enzymes. Recently, an attempt was made to remineralize the demineralized collagen matrix of caries-affected dentin with meta-stabilized amorphous calcium phosphate. Kim et al. [11], in a study on the relationship between resin-dentin bonding and collagen fiber remineralization, showed that remineralization of dentin collagen fibers using calcium phosphate ion clusters (CPIC) and metastable calcium phosphate solutions increased dentin bond strength.

The purpose of this study was to evaluate the effects of flavonoids, CPIC, and flavonoids combined with CPIC on dentin bonding stability through bond strength and nanoleakage assessments. The null hypothesis tested was that dentin treatment with flavonoids and CPIC during the bonding process would not affect dentin bond strength and nanoleakage at the resin-dentin interfaces. This study tested the feasibility of using three different flavonoids, which have not been used in the dental field, to improve the stability of the resin-dentin interfaces. In addition, we evaluated the effects of combinations of flavonoids and CPIC on resin-dentin bonding for the first time.

## 2. Materials and Methods

### 2.1. Formulation of Experimental Solutions

#### 2.1.1. Preparation of Flavonoid Solutions

**Figure 1:**
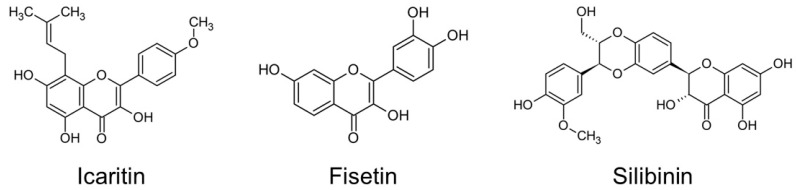
Chemical structure of flavonoids used in this study.

The structure and physicochemical properties of the flavonoids used in this study are shown in Figure 1 and Table 1. Three flavonoid solutions incorporating icaritin (ICT), fisetin (FIS), and silibinin (SIB) were synthesized. ICT and SIB have a proanthocyanidin monomeric unit as their basic structure, and FIS is a 3-hydroxyflavone with a Jaceosidin structure. To obtain the maximum solubility without affecting the properties of each flavonoid, a flavonoid solution was generated using an exclusive equation (Table 2) [16,17].

#### 2.1.2. Preparation of a CPIC Solution

CPIC was synthesized according to the method proposed by Shao et al. [18]. The following chemicals were used without further purification: calcium chloride dihydrate (CaCl_2_·2H_2_O 99.0%, Sigma-Aldrich, St. Louis, MO, USA), triethylamine (TEA; (C_2_H_5_)_3_N 99.5%, Sigma-Aldrich), phosphoric acid (H_3_PO_4_ 85% in H_2_O solution, Sigma-Aldrich), and ethyl alcohol (C_2_H_5_OH, Samchun, Seoul, Korea).

Two solutions were prepared for the synthesis of the CPIC. For solution A, 0.20 g of calcium chloride dihydrate and 3.8 mL of TEA were added to 80 mL of ethanol and ultrasonicated for 5 min. For solution B, 70 μL of phosphoric acid was added to 20 mL of ethanol and mixed thoroughly. A CPIC solution (2 mg/mL) was generated by dropping solution B into solution A with minimal agitation.

#### 2.1.3. Preparation of Flavonoid and CPIC Combination Solutions

Flavonoid and CPIC combination solutions were prepared by gently mixing 200 µL of each of the three flavonoid solutions (ICT, FIS, and SIB) and 200 µL of CPIC solution in a 1.5 mL microcentrifuge tube in a 1:1 ratio.

Three flavonoid solutions (ICT, FIS, and SIB), CPIC solution (CPIC), and three flavonoid and CPIC combination solutions (ICT + C, FIS + C, and SIB + C) were used for dentin treatment. The group in which no experimental solution was applied was used as the control group (CON) (Table 3).

### 2.2. Dentin Specimen Preparation

Sixty-four extracted premolars or molars from patients aged 18–35 years were used in this study. The study protocol was approved by the Institutional Review Board of Pusan National University Dental Hospital (Yangsan, Republic of Korea) under the approval number PNUDH-2021-011. The teeth were disinfected with 0.5% chloramine, stored in distilled water, and used within 3 months of extraction. The coronal enamel of the teeth was cut in a direction parallel to the occlusal plane using a low-speed diamond saw (Accutom-100, Struers, Cleveland, OH, USA) to expose the dentin surface. The smear layer was produced using 320- and 600-grit SiC paper under water irrigation. The dentin surface was etched with Ultra Etch (35% H_3_PO_4_; Ultradent, South Jordan, UT, USA) for 15 s. Then, it was rinsed with distilled water for 30 s and gently air-dried for 5 s. The teeth were randomly allocated to one of the experimental groups (*n* = 8). Each experimental solution, except for the CON group, was applied to the dentin surface for 1 min and slightly air-dried for 2 s. If necessary, the excess solution was removed with an absorbent paper, and the dentin surface was kept wet.

### 2.3. Fourier Transform Infrared Spectroscopy

The dentin surface pretreated with the experimental solution was analyzed using Fourier transform infrared (FTIR) spectroscopy (Spectrum GX, Perkin Elmer Corp., Waltham, MA, USA) at 4 cm^−1^ resolution and 32 scans in transmittance mode. The spectral range was 2000–400 cm^−1^ in order to survey peaks and shoulders at 1633 cm^−1^ (amide I), 1544 cm^−1^ (amide II), 1450 cm^−1^ (CH_2_ bending), and 1235 cm^−1^ (amide III) assigned to dentin collagen cross-linking after normalization and baseline correction processes. The analysis was performed in triplicates.

### 2.4. Microtensile Bond Strength Testing

**Figure 2:**
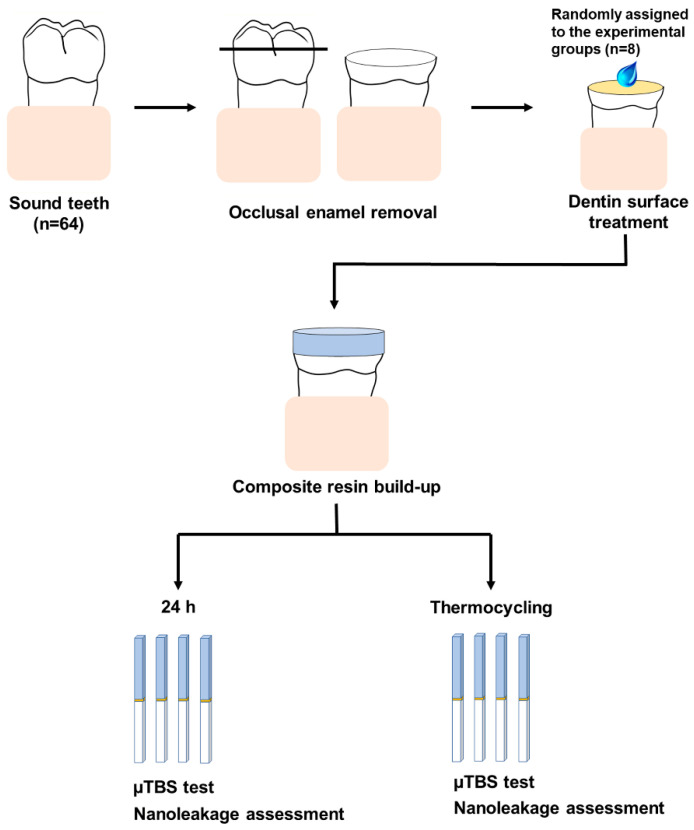
Schematic diagram illustrating the procedures for preparing the bonded specimens used in the microtensile bond strength and nanoleakage tests.

The experimental procedures for the fabrication of bonded tooth specimens for the microtensile strength and nanoleakage tests are illustrated in Figure 2. A universal adhesive (All-Bond Universal, BISCO Inc., Schaumburg, IL, USA) was applied to the pretreated dentin surface according to the manufacturer’s instructions and light-cured for 10 s with an LED curing unit (Valo, Ultradent Products, South Jordan, UT, USA). Composite resin (Z350, 3M ESPE, Monrovia, CA, USA) was built up to 5 mm in increments of approximately 1 mm. Each increment was light-cured for 40 s.

One half of the bonded tooth specimens was stored in distilled water at 37 °C for 24 h, and the other half was subjected to 10,000 thermocycles (dwell time of 30 s from 5 °C to 55 °C, transfer time 5 s. After storage, four bonded tooth specimens for each subgroup were longitudinally sectioned, perpendicular to the bonded interface, into resin-dentin sticks with a cross-sectional area of 1.0 mm^2^ using a low-speed diamond saw (Accutom-100, Struers, Cleveland, OH, USA). Approximately 6–9 bonded sticks were obtained per tooth. Among them, two sticks for each subgroup were assigned to the nanoleakage assessment. The microtensile bond strength (µTBS) values were measured using a µTBS tester at a cross-head speed of 1 mm/min.

All fractured specimens were examined using a stereomicroscope (40× magnification; SZH-131, Olympus, Tokyo, Japan) to identify the failure modes. The failure modes were classified as adhesive (failure at resin-dentin interface), cohesive in dentin (failure exclusive within dentin), cohesive in composite (failure exclusive within resin composite), or mixed (failure at resin-dentin interface that included cohesive failure of the adjacent substrates).

### 2.5. Nanoleakage Assessment

Two resin-dentin sticks from each subgroup were used for the nanoleakage assessment. The specimens were immersed in ammoniacal silver nitrate solution, blocked from light for 24 h, and then washed with distilled water [19]. The specimens were then immersed in a photo-developing solution under fluorescent light for 8 h. After embedding in epoxy resin, they were polished using 4000-grit SiC paper (Buehler, Coventry, UK). After dehydration and drying, the specimens were inspected using a scanning electron microscope (SEM; JSM-7900F, JEOL, Peabody, MA, USA).

### 2.6. Statistical Analysis

The µTBS data were analyzed using one-way analysis of variance (ANOVA), followed by Tukey’s test for multiple comparisons. The data were analyzed using a statistical software (R language program, R Foundation for Statistical Computing, Vienna, Austria). The level of significance was set at α = 0.05.

## 3. Results

The results of the µTBS tests at 24 h and after thermocycling are summarized in Table 4. A one-way ANOVA revealed significant differences among groups at 24 h and after thermocycling (*p* < 0.01). According to the post hoc analysis, FIS (26.81 ± 4.56 MPa) and ICT + C (30.63 ± 5.49 MPa) showed significantly higher µTBS than CON (21.66 ± 3.47 MPa) at 24 h. CPIC (23.43 ± 3.37 MPa), ICT + C (26.74 ± 6.01 MPa), FIS + C (23.42 ± 4.10 MPa), and SIB + C (25.17 ± 4.08 MPa) showed significantly higher µTBS than CON (19.18 ± 4.96 MPa) after thermocycling (*p* < 0.01). ICT + C showed the highest mean values at 24 h and after thermocycling. The comparisons between 24 h and thermocycling within each group revealed that there was no statistically significant decrease in µTBS for FIS + C and SIB + C after thermocycling, and their µTBS values were significantly higher than those of CON (*p* < 0.01).

**Figure 3:**
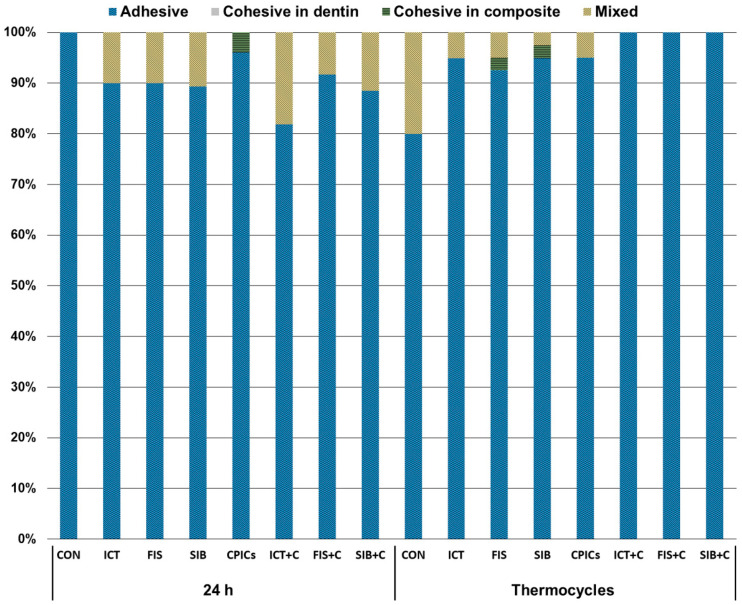
Distribution of failure modes after the microtensile bond strength test.

**Figure 4:**
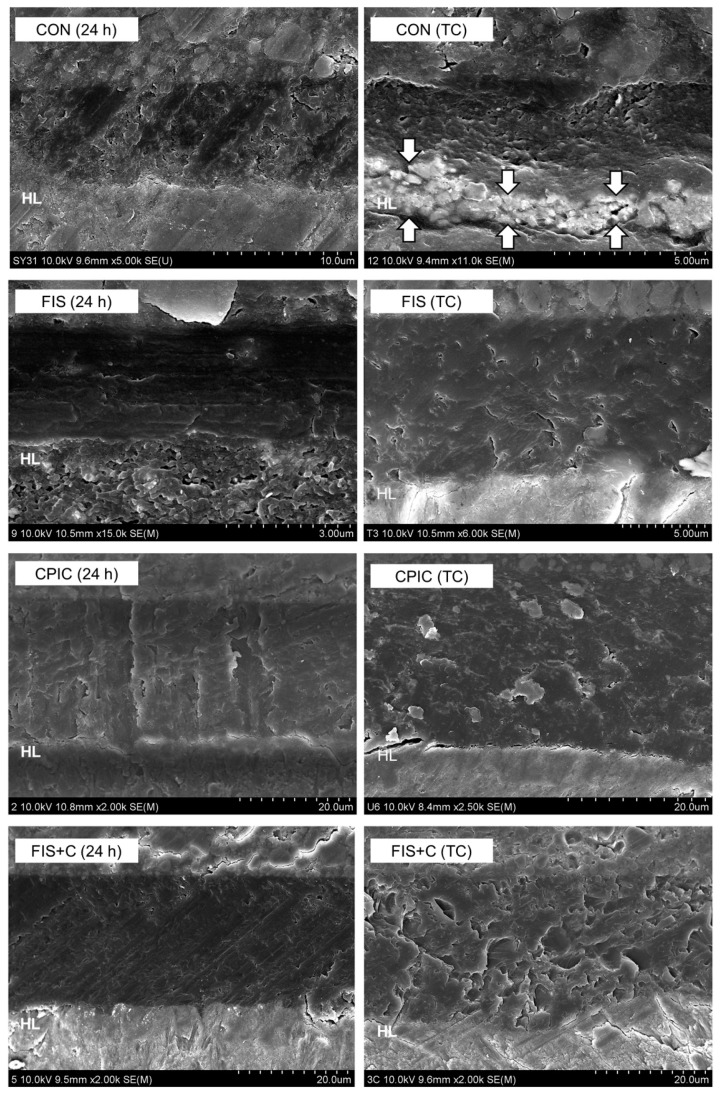

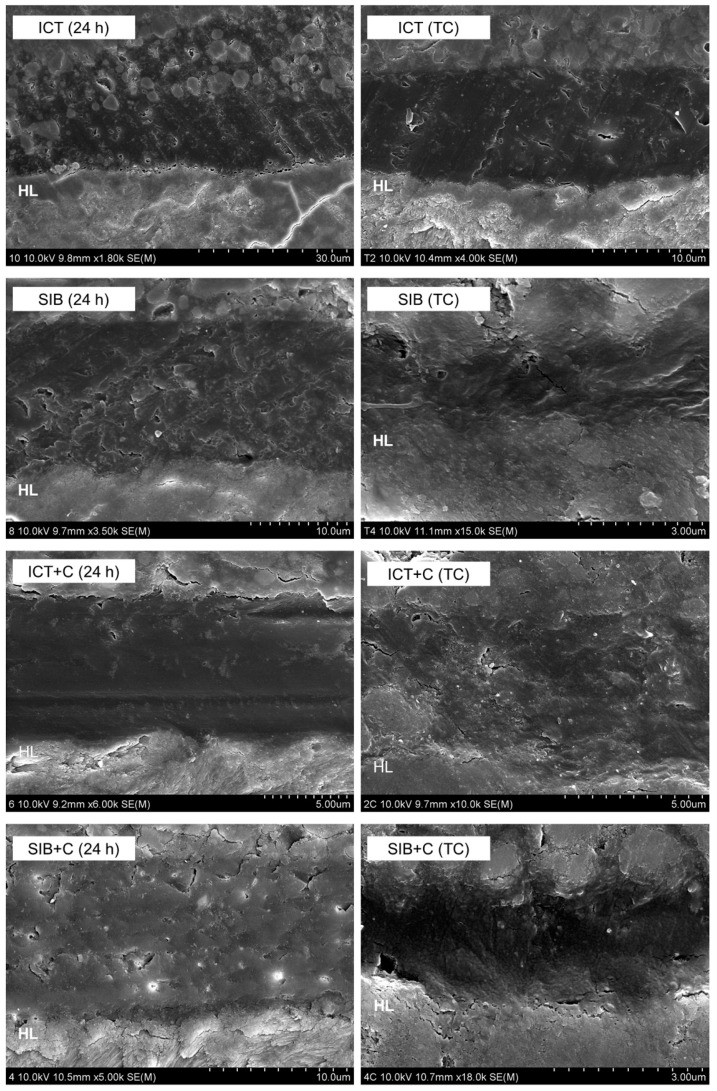
(continued on next page). Representative SEM images of the resin-dentin interfaces at 24 h and after thermocycling (TC). The structure marked with HL represents the hybrid layers. In contrast to the other group, the control group (CON) exhibited noticeable silver infiltration (arrows) throughout the hybrid layer after TC. ICT: icaritin; FIS: fisetin; SIB; silibinin; CPIC: calcium phosphate ion clusters; ICT + C: icaritin and CPIC combination; FIS + C: fisetin and CPIC combination; SIB + C: silibinin and CPIC combination.

The distribution of the failure modes is shown in Figure 3. The predominant failure mode was adhesive failure in all of the groups. The groups incorporating flavonoids showed mixed and cohesive failures, even after thermocycling. Representative SEM images of the resin–dentin interfaces are shown in Figure 4. After thermocycling, silver infiltration into the hybrid layer and interfacial gaps were more noticeable in CON than in the other groups.

**Figure 5:**
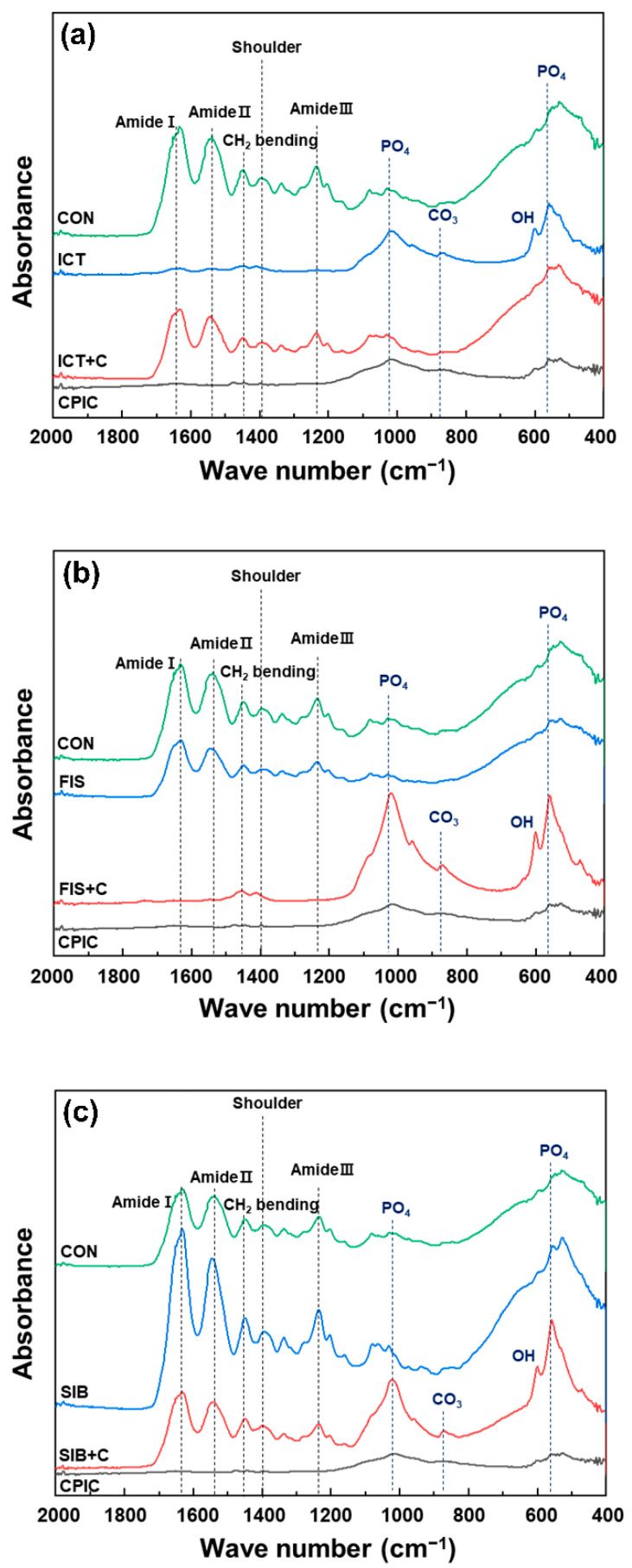
Fourier transform infrared spectroscopy spectra of dentin specimens conditioned with the experimental solutions in comparison with the control group (CON): (**a**) icaritin (ICT) and icaritin combined with CPIC (ICT + C); (**b**) fisetin (FIS) and fisetin combined with CPIC (FIS + C); (**c**) silibinin (SIB) and silibinin combined with CPIC (SIB + C). The flavonoid and CPIC combination groups (ICT + C, FIS + C, and SIB + C) exhibited notable changes in the emergence of chemical functional group spectra characteristic of apatitic materials, including absorption bands of PO_4_^3−^ antisymmetric stretching mode (ν3) at 1080–1000 cm^−1^, PO_4_^3−^ antisymmetric bending mode (ν4) at 570 cm^−1^, OH^−^ at 631 cm^−1^, and CO_3_^2−^ at 875 cm^−1^.

The FTIR spectra of the specimens treated with the experimental solutions are shown in Figure 5. The amide-related peaks and shoulders were revealed at 1633 cm^−1^ (amide I), 1544 cm^−1^ (amide II), and 1235 cm^−1^ (amide III). The peak at 1450 cm^−1^ was attributed to the CH_2_ bending, and these peaks were assigned to collagen cross-linking [20,21]. The peaks at 1029, 860, and 557 cm^−1^ were related to dentin phosphate [22]. The flavonoid and CPIC combination groups exhibited notable changes in the emergence of chemical functional group spectra characteristic of non-stoichiometric hydroxyapatite compared to CON. These included absorption bands of the PO_4_^3−^ antisymmetric stretching mode (ν3) at 1080–1000 cm^−1^, PO_4_^3−^ antisymmetric bending mode (ν4) at 570 cm^−1^, OH^−^ at 631 cm^−1^, and CO_3_^2−^ at 875 cm^−1^ [23].

## 4. Discussion

In this study, we investigated the potential effects of flavonoids and CPIC on dentin bonding stability. Dentin treatment with flavonoids and CPIC solutions improved the integrity of the resin-dentin interface. Additionally, the flavonoid and CPIC combination groups presented an improved bonding stability with significantly higher bond strengths after aging compared to the control group.

Despite the continuous development of dental adhesive systems, dentin bonding still has weaknesses at the resin-dentin interfaces owing to the unique organic properties of dentin [1,2]. The degradation of resin–dentin bonds is a complex process in which both resin and collagen components are hydrolyzed. More than 90% of the extracellular matrix (ECM) in dentin is composed of type I collagen fibers. The stability of the hybrid layer is directly affected by the integrity of the collagen fibers. However, collagen fibrils are exposed by acid etching during bonding procedures and are not completely infiltrated by resin monomers [5]. These unfilled porosities lead to a nanoleakage, which acts as a channel for water sorption and the leaching of uncured acidic monomers. Water and bacterial oral fluids absorbed through nanoleakage facilitate MMP activity and participate in hydrolysis of collagen fibrils [4,24]. The collagen fibrils are broken down and their mass is replaced by water. The increase in nanoleakage can be considered as a degradation of collagen fibrils in the hybrid layer [25]. Thus, silver nitrate penetration indicates an increase in water absorption that impairs dentin bonding stability [26].

Unprotected collagen fibers which are exposed during dentin demineralization are vulnerable to biodegradation by MMPs [2,7,8]. MMPs consist of about 30 types of calcium-dependent zinc endopeptidases that degrade ECM [8,27]. MMP-1 and -8 (collagenous), -2 and -9 (gelatinase), and -3 (stromelysin) are found in dentin. MMPs are usually secreted into the ECM in the form of inactive proenzymes and degrade matrix components in low-pH environments [24]. MMPs can be exposed and activated by self-etch and etch-and-rinse adhesive systems during bonding procedures. Demineralized dentin contains MMP-2, -3, -8, -9, and -20 and cathepsins 4 and 5 in active forms [8].

Enzymatic inactivation methods that inhibit the destruction of the dentinal organic matrix have been studied to improve the stability and durability of the resin–dentin interfaces [8,10,27,28]. One of the mechanisms by which MMPs degrade collagen networks is that this protease unwinds collagen chains when it binds to collagen molecules. This process depends on the fact that the active site of MMPs has enough space for a specific glycine-isoleucine peptide of collagen polypeptide chains to bind to. The application of cross-linking agents to dentin can stiffen the collagen polypeptide chain so that it does not unwind, or it can create new peptide bonds across adjacent peptides and inactivate the catalytic site of a protease [14]. Some researchers examined the effect of flavonoids as natural cross-linking agents for improving dentin bonding stability [12,15,16,17,21,29]. A biomimetic remineralization system using CPIC and metastable calcium phosphate solutions was proposed as another method to improve the stability and durability of the dentin collagen matrix [11]. It was shown that the dentin bond strength increased and collagen remineralization was induced by applying CPIC or a metastable calcium phosphate solution to the demineralized dentin matrix.

This study included three flavonoids-icaritin, fisetin, and silibinin-as potential cross-linking agents. Icaritin is a flavonoid glycoside isolated from the herb, Epimedii. According to studies on human bone marrow and human adipose tissue-derived mesenchymal stem cells, icaritin has an estrogen-like activity [30,31], improves osteoblast proliferation and differentiation, inhibits osteoclast activity, and promotes bone matrix calcification [32,33]. Fisetin is a flavonoid extracted from the flavonoid group of polyphenols. Fisetin has an antiproliferative effect, inducing apoptosis activation, and produces an inhibitory effect on inflammatory cytokine activity [34]. Silibinin, the main active ingredient in silymarin extracted from milk thistle seeds, has antioxidant and antihepatotoxic effects [35]. All three flavonoids have inhibitory effects on MMP-2 and MMP-9 as a natural cross-linking agent [36,37,38]. However, there is a lack of studies on the effects of these three flavonoids on dentin bonding stability; therefore, our study evaluated these flavonoids as potential cross-linking agents for dentin biomodification.

Previous studies on the effects of various flavonoids on dentin bonding reported conflicting results [12,16,17,29]. Fang et al. [17] reported that proanthocyanidin improved the bond strength even with a short treatment duration of 60 s. In contrast, Dávila-Sánchez et al. [16] found an improvement in bond strength with quercetin, naringin, and rutin, but there was no improvement with hesperidin and proanthocyanidin. The molecular characteristics of flavonoids, concentration, and treatment time can influence the effect of flavonoids as a collagen cross-linker [16,17]. Among the flavonoids tested in the present study, fisetin significantly improved the immediate µTBS value compared to the other flavonoids and the control groups. This result corresponds with an earlier study that reported that the interaction between the cross-linking agents and dentin depends on their ability to form covalent or hydrogen bonds with collagen fibers [15]. The number of hydroxyphenyl groups and the molecular size of a flavonoid affect the ability to form covalent or ionic bonds with hydroxyl, carboxyl, amine, and amide groups in collagen. In addition, the effect of improving the mechanical properties of the dentin collagen matrix is related to the solubility index of the cross-linking agent and the number of molecules available in the solution [16]. The improved immediate µTBS value for FIS can be explained by the fact that fisetin has a low molecular weight, the highest precision value of a hydroxyphenyl group, and the highest solubility among the flavonoids tested.

Conversely, FIS showed a significant decrease in µTBS after thermocycling, but its value was comparable to that of CON. Fisetin is similar to quercetin in its molecular structure and the number of hydroxyphenyl and alcoholic radicals. The decreased µTBS value after thermocycling is presumed to be due to the weakening of the cross-linking effect in the hybrid layer by aging, since a flavonoid with the quercetin structure does not have a glycoside moiety that is resistant to an acidic environment [16].

Although the flavonoids did not exhibit a significantly improved bond strength after thermocycling, they contributed to improving the quality of the resin–dentin interfaces, as shown by the reduced nanoleakage. The control group showed noticeable silver infiltration and interfacial gaps within the hybrid layer, whereas the groups incorporating flavonoids showed less silver infiltration and voids even after aging. These results are in accordance with previous findings that cross-linking agents have the ability to control the level of residual water bound to the ECM of dentin, improve adhesive infiltration, and prevent the degradation of the cross-linking network [14].

The flavonoid and CPIC combination groups (ICT + C, FIS + C, and SIB + C) and the CPIC group showed significantly higher µTBS values than CON after thermocycling. The demineralized dentin collagen fibers should be encapsulated by adhesive resin for stable bonding, but this is difficult to achieve in real practical conditions because of residual water and the discrepancy between the depth of etching and resin infiltration [2]. The effect of CPIC on improving dentin bonding stability is based on the fact that MMPs are unable to hydrolyze collagen in the absence of water [11]. CPIC induce the growth of apatite crystals, which are used as collagen-binding scaffolds, and inhibit MMPs by replacing residual water in demineralized collagen fibers with new apatite minerals [11,39]. The increased µTBS values are explained by the fact that CPIC treatment induced the remineralization of the acid-etched dentin substrate. This remineralization improved the mechanical properties and durability of the hybrid layer. The FTIR results also revealed the formation of apatitic minerals in the demineralized dentin in the CPIC-incorporating groups.

This study has some limitations. The flavonoid and CPIC combinations were mixed at a ratio of 1:1, but no research was conducted to determine whether the collagen remineralization effect would be different depending on the mixing ratio. In addition, we did not directly evaluate the inhibitory effects of flavonoids and CPIC on MMPs. According to a study by Kim et al. [11], the authors expected that the MMP inhibitory effect would be proportional to the CPIC concentration, but it was not dependent on the concentration of CPIC. Therefore, further studies are required to determine the optimal mixing ratio of flavonoids and CPIC to improve the resin–dentin bonding stability.

## 5. Conclusions

This study demonstrated that the combinations of the flavonoids and CPIC improved the resin–dentin bond strength after the artificial aging by thermocycling. ICT + C showed the highest bond strength values both at 24 h and after thermocycling. FIS + C and SIB + C also maintained higher bond strengths after thermocycling as compared to the control group. In the aspect of structural integrity of the resin-dentin interfaces, the flavonoid and the CPIC combination groups showed less nanoleakage than the control group.

Within the limitations of the present study, the pretreatment of flavonoids and flavonoids combined with CPIC on demineralized dentin was effective for improving dentin bonding stability. The biomodification of dentin substrates is a feasible strategy to overcome the limitations of the current adhesive systems.

## Figures and Tables

**Table 1:** Physical and chemical properties of flavonoids used in this study.

Substance	Molecular Mass	Number of Mols (6.5% mass)	Solubility
Icaritin (ICT)	368.38 g/mol	1.76 mM	0.00821 mg/mL in water
Fisetin (FIS)	286.24 g/mol	2.27 mM	5 mg/mL in ethanol
Silibinin (SIB)	482.44 g/mol	1.35 mM	<0.04 mg/mL in water

**Table 2:** Composition of a hydroalcoholic solution of flavonoid (6.5% mass).

Component	Compound	Quantity %
Active compound	Flavonoid	6.5% mass
Vehicle	Pure ethanol	30% (3 mL)
Surfactant	Span^®^ 20 (sorbitan monolaurate)	1% (0.1 mL)
Aqueous medium	Distilled water	QS 10 mL

**Table 3:** Experimental groups used in this study.

Groups	Dentin Treatment Solutions
CON	No application of experimental solution
ICT	Icaritin
FIS	Fisetin
SIB	Silibinin
CPIC	Calcium phosphate ion clusters
ICT + C	Icaritin + CPIC
FIS + C	Fisetin + CPIC
SIB + C	Silibinin + CPIC

**Table 4:** Mean (SD) microtensile bond strength values in MPa of the experimental groups.

Groups	24 h	Thermocycling
CON	21.66 (3.47) ^C^	19.18 (4.96) ^c,^*
ICT	24.40 (4.63) ^BC^	20.53 (3.09) ^bc,^*
FIS	26.81 (4.56) ^AB^	19.40 (4.84) ^c,^*
SIB	25.65 (4.41) ^BC^	22.04 (4.79) ^bc,^*
CPIC	25.97 (4.03) ^BC^	23.43 (3.37) ^ab,^*
ICT + C	30.63 (5.49) ^A^	26.74 (6.01) ^a,^*
FIS + C	25.63 (4.25) ^BC^	23.42 (4.10) ^ab^
SIB + C	24.76 (3.67) ^BC^	25.17 (4.08) ^a^

Different superscript letters within each column indicate statistically significant differences (uppercase letters for 24 h and lowercase letters for thermocycling); * There were significant differences in mean values between 24 h and thermocycling.

## Data Availability

The data presented in this study are available on request from the corresponding authors.

## References

[1] Van Meerbeek B., Inokoshi S., Braem M., Lambrechts P., Vanherle G. (1992). Morphological aspects of the resin-dentin interdiffusion zone with different dentin adhesive systems. J. Dent. Res..

[2] Pashley D.H., Tay F.R., Breschi L., Tjäderhane L., Carvalho R.M., Carrilho M., Tezvergil-Mutluay A. (2011). State of the art etch-and-rinse adhesives. Dent. Mater..

[3] Nakabayashi N., Ashizawa M., Nakamura M. (1992). Identification of a resin-dentin hybrid layer in vital human dentin created in vivo: Durable bonding to vital dentin. Quintessence Int..

[4] Hashimoto M., Ohno H., Kaga M., Endo K., Sano H., Oguchi H. (2000). In vivo degradation of resin-dentin bonds in humans over 1 to 3 years. J. Dent. Res..

[5] Sano H., Shono T., Takatsu T., Hosoda H. (1994). Microporous dentin zone beneath resin-impregnated layer. Oper. Dent..

[6] Bourbia M., Finer Y. (2018). Biochemical stability and interactions of dental resin composites and adhesives with host and bacteria in the oral cavity: A review. J. Can. Dent. Assoc..

[7] Tjäderhane L., Larjava H., Sorsa T., Uitto V.J., Larmas M., Salo T. (1998). The activation and function of host matrix metalloproteinases in dentin matrix breakdown in caries lesions. J. Dent. Res..

[8] Perdigão J., Reis A., Loguercio A.D. (2013). Dentin adhesion and MMPs: A comprehensive review. J. Esthet. Restor. Dent..

[9] Dos Santos P.H., Karol S., Bedran-Russo A.K. (2011). Long-term nano-mechanical properties of biomodified dentin-resin interface components. J. Biomech..

[10] Tjäderhane L., Nascimento F.D., Breschi L., Mazzoni A., Tersariol I.L., Geraldeli S., Tezvergil-Mutluay A., Carrilho M.R., Carvalho R.M., Tay F.R. (2013). Optimizing dentin bond durability: Control of collagen degradation by matrix metalloproteinases and cysteine cathepsins. Dent. Mater..

[11] Kim H., Choi A., Gong M.K., Park H.R., Kim Y.I. (2020). Effect of remineralized collagen on dentin bond strength through calcium phosphate ion clusters or metastable calcium phosphate solution. Nanomaterials.

[12] Islam M.S., Hiraishi N., Nassar M., Yiu C., Otsuki M., Tagami J. (2014). Effect of hesperidin incorporation into a self-etching primer on durability of dentin bond. Dent. Mater..

[13] Kumar S., Pandey A.K. (2013). Chemistry and biological activities of flavonoids: An overview. Sci. World J..

[14] Leme-Kraus A.A., Aydin B., Vidal C.M., Phansalkar R.M., Nam J.W., McAlpine J., Pauli G.F., Chen S., Bedran-Russo A.K. (2017). Biostability of the proanthocyanidins-dentin complex and adhesion studies. J. Dent. Res..

[15] He L., Mu C., Shi J., Zhang Q., Shi B., Lin W. (2011). Modification of collagen with a natural cross-linker, procyanidin. Int. J. Biol. Macromol..

[16] Dávila-Sánchez A., Gutierrez M.F., Bermudez J.P., Méndez-Bauer M.L., Hilgemberg B., Sauro S., Loguercio A.D., Arrais C.A.G. (2020). Influence of flavonoids on long-term bonding stability on caries-affected dentin. Dent. Mater..

[17] Fang M., Liu R., Xiao Y., Li F., Wang D., Hou R., Chen J. (2012). Biomodification to dentin by a natural crosslinker improved the resin–dentin bonds. J. Dent..

[18] Shao C., Jin B., Mu Z., Lu H., Zhao Y., Wu Z., Yan L., Zhang Z., Zhou Y., Pan H. (2019). Repair of tooth enamel by a biomimetic mineralization frontier ensuring epitaxial growth. Sci. Adv..

[19] Tay F.R., Pashley D.H., Yoshiyama M. (2002). Two modes of nanoleakage expression in single-step adhesives. J. Dent. Res..

[20] Liu Y., Bai X., Li S., Liu Y., Keightley A., Wang Y. (2015). Molecular weight and galloylation affect grape seed extract constituents’ ability to cross-link dentin collagen in clinically relevant time. Dent. Mater..

[21] Liu Y., Chen M., Yao X., Xu C., Zhang Y., Wang Y. (2013). Enhancement in dentin collagen’s biological stability after proanthocyanidins treatment in clinically relevant time periods. Dent. Mater..

[22] Yin J., Mei M.L., Li Q., Xia R., Zhang Z., Chu C.H. (2016). Self-cleaning and antibiofouling enamel surface by slippery liquid-infused technique. Sci. Rep..

[23] Berzina-Cimdina L., Borodajenko N., Theophile T. (2012). Research of calcium phosphate using Fourier transform infrared spectroscopy. Infrared Spectroscopy—Materials Science, Engineering and Technology.

[24] Carrilho M.R., Tay F.R., Donnelly A.M., Agee K.A., Tjäderhane L., Mazzoni A., Breschi L., Foulger S., Pashley D.H. (2009). Host-derived loss of dentin matrix stiffness associated with solubilization of collagen. J. Biomed. Mater. Res. B Appl. Biomater..

[25] Carrilho M.R., Geraldeli S., Tay F., de Goes M.F., Carvalho R.M., Tjäderhane L., Reis A.F., Hebling J., Mazzoni A., Breschi L. (2007). In vivo preservation of the hybrid layer by chlorhexidine. J. Dent. Res..

[26] Breschi L., Martin P., Mazzoni A., Nato F., Carrilho M., Tjäderhane L., Visintini E., Cadenaro M., Tay F.R., De Stefano Dorigo E. (2010). Use of a specific MMP-inhibitor (galardin) for preservation of hybrid layer. Dent. Mater.

[27] Li H., Li T., Li X., Zhang Z., Li P., Li Z. (2015). Morphological effects of MMPs inhibitors on the dentin bonding. Int. J. Clin. Exp. Med..

[28] Scheffel D.L., Hebling J., Scheffel R.H., Agee K., Turco G., de Souza Costa C.A., Pashley D. (2014). Inactivation of matrix-bound matrix metalloproteinases by cross-linking agents in acid-etched dentin. Oper. Dent..

[29] De-Paula D.M., Lomonaco D., Ponte A.M.P., Cordeiro K.E., Moreira M.M., Mazzetto S.E., Feitosa V.P. (2020). Influence of collagen cross-linkers addition in phosphoric acid on dentin biomodification and bonding of an etch-and-rinse adhesive. Dent. Mater..

[30] Wong S.P., Shen P., Lee L., Li J., Yong E.L. (2009). Pharmacokinetics of prenylflavonoids and correlations with the dynamics of estrogen action in sera following ingestion of a standardized Epimedium extract. J. Pharm. Biomed. Anal..

[31] Wang Z.Q., Lou Y.J. (2004). Proliferation-stimulating effects of icaritin and desmethylicaritin in MCF-7 cells. Eur. J. Pharmacol..

[32] Yao D., Wang X., Xie X., Zhang G., Qin L. (2010). Icaritin promotes osteogenic differentiation while inhibits osteoclastic differentiation in vitro. Bone.

[33] Long J., Zhou Q., Li D., Wang X., Cao H., Qin L. (2014). Phytoestrogenic molecule icaritin prevents OVX-induced osteoporosis in mice. J. Orthop. Translat..

[34] Gupta S.C., Tyagi A.K., Deshmukh-Taskar P., Hinojosa M., Prasad S., Aggarwal B.B. (2014). Downregulation of tumor necrosis factor and other proinflammatory biomarkers by polyphenols. Arch. Biochem. Biophys..

[35] Bhatia N., Zhao J., Wolf D.M., Agarwal R. (1999). Inhibition of human carcinoma cell growth and DNA synthesis by silibinin, an active constituent of milk thistle: Comparison with silymarin. Cancer Lett..

[36] Wang X.F., Wang J. (2014). Icaritin suppresses the proliferation of human osteosarcoma cells in vitro by increasing apoptosis and decreasing MMP expression. Acta Pharmacol. Sin..

[37] Park J.H., Jang Y.J., Choi Y.J., Jang J.W., Kim J.H., Rho Y.K., Kim I.J., Kim H.J., Leem M.J., Lee S.T. (2013). Fisetin inhibits matrix metalloproteinases and reduces tumor cell invasiveness and endothelial cell tube formation. Nutr. Cancer.

[38] Oh S.J., Jung S.P., Han J., Kim S., Kim J.S., Nam S.J., Lee J.E., Kim J.H. (2013). Silibinin inhibits TPA-induced cell migration and MMP-9 expression in thyroid and breast cancer cells. Oncol. Rep..

[39] Wang J., Chen Y., Li L., Sun J., Gu X., Xu X., Pan H., Tang R. (2013). Remineralization of dentin collagen by meta-stabilized amorphous calcium phosphate. CrystEngComm.

